# Screening and characterization of lactic acid bacteria and fermentation of gamma-aminobutyric acid-enriched bamboo shoots

**DOI:** 10.3389/fmicb.2024.1333538

**Published:** 2024-02-05

**Authors:** Meilin Chen, Hongqiu Xia, Xifeng Zuo, Danping Tang, Haoyu Zhou, Zijun Huang, Ailing Guo, Jun Lv

**Affiliations:** ^1^College of Food Science and Technology, Huazhong Agriculture University, Wuhan, Hubei, China; ^2^Liunan District Modern Agricultural Industry Service Center of Liuzhou City, Liuzhou, Guangxi, China; ^3^Institute of Infection and Immunity, Taihe Hospital, Hubei University of Medicine, Shiyan, China

**Keywords:** bamboo shoots, lactic acid bacteria, GABA, fermentation, inoculation

## Abstract

In order to produce fermented bamboo shoots with functional properties, two strains of lactic acid bacteria were selected for inoculation and fermentation. One strain, *Lactiplantibacillus plantarum* R1, exhibited prominent potential probiotic properties (including gastrointestinal condition tolerance, adhesion ability, antimicrobial ability, and antibiotic resistance), while the other, *Levilactobacillus brevis* R2, demonstrated the capability of high γ-aminobutyric acid (GABA) production (913.99 ± 14.2 mg/L). The synergistic inoculation of both strains during bamboo shoot fermentation led to a remarkable increase in GABA content (382.31 ± 12.17 mg/kg), surpassing that of naturally fermented bamboo shoots by more than 4.5 times and outperforming mono-inoculated fermentation. Simultaneously, the nitrite content was maintained at a safe level (5.96 ± 1.81 mg/kg). Besides, inoculated fermented bamboo shoots exhibited an increased crude fiber content (16.58 ± 0.04 g/100 g) and reduced fat content (0.39 ± 0.02 g/100 g). Sensory evaluation results indicated a high overall acceptability for the synergistically inoculated fermented bamboo shoots. This study may provide a strategy for the safe and rapid fermentation of bamboo shoots and lay the groundwork for the development of functional vegetable products enriched with GABA.

## 1 Introduction

Bamboo shoots are known as the “King of Forest Vegetables” due to their low-fat content and high levels of dietary fiber, protein and minerals. However, the challenge with fresh bamboo shoots is their rapid lignification after harvesting (Zhang et al., 2020), which significantly reduces their food quality and commercial value. To address this issue, various processing methods are employed, including fermentation, drying, steaming, and pickling. Out of these methods, fermentation leads to significant changes in the content of amino acids, organic acids, aromatic compounds, esters, and other substances, resulting in a unique flavor, which has contributed to the growing popularity of fermented bamboo shoots.

Similar to most fermented vegetables, the fermentation process of bamboo shoots is significantly influenced by lactic acid bacteria (LAB), which include various species such as *Lactobacillus* sp., *Leuconostoc* sp., *Weissella* sp., and *Pediococcus* sp. (Guan et al., 2020; Chen C. et al., 2022; Li et al., 2022; Das et al., 2023). Indeed, numerous studies have demonstrated the intricate connection between complex microbial communities and the production of distinctive flavor compounds in fermented bamboo shoots. Substances like p-cresol, lactic acid, acetic acid, 1-octen-3-one, and others contribute to the unique and characteristic flavors developed during the fermentation process (Chen C. et al., 2022; Tang et al., 2024). However, the unpredictable microbial succession and challenging-to-control fermentation environment can lead to quality issues, including the formation of undesirable components, off-flavors, and undesirable coloration. In response to these challenges, researchers have proposed the method of inoculated fermentation. *Lactiplantibacillus plantarum* stands out as one of the most commonly employed strains in inoculated fermentation. It demonstrates effective inhibition of nitrite and pathogenic bacteria, while also enhances the overall flavor of products (Zhang et al., 2022; Yu et al., 2023). Additionally, other strains such as *Levilactobacillus brevis*, *Lactobacillus sake*, and *Leuconostoc mesenteroides* have also shown positive impacts on the fermentation food (Kim et al., 2017; Xia et al., 2017; Huang et al., 2021). Furthermore, researchers discovered that employing multiple strains for synergistic fermentation not only enhances the safety of the fermentation process but also contributes to a more robust flavor profile (Luo et al., 2020; Hu et al., 2021). The inoculation of *Leuconostoc citreum* and *L. plantarum* for bamboo shoots fermentation demonstrated significant inhibition of the growth of *Enterococcus* and *Clostridium*, suggesting potential benefits. Moreover, the inoculation ratio of the two strains had a notable impact on the flavor compounds after fermentation (Lu et al., 2022). However, there is limited research on the application of synergistic inoculation fermentation in bamboo shoots, and further investigations are warranted on different LAB strains and their effects on the nutrition and flavor.

Gamma-aminobutyric acid (GABA) is a non-protein amino acid widely distributed in plants, animals and microorganisms. It is synthesized through the decarboxylation of L-glutamic acid (L-Glu) catalyzed by the enzyme glutamic acid decarboxylase (GAD, EC 4.1.1.15). Serving as an important inhibitory neurotransmitter, GABA has a wide range of physiological functions, such as improving sleep, treating diabetes, alleviating anxiety, and combating obesity (Pannerchelvan et al., 2023). Compared to GABA obtained from plant and animal sources, microbial fermentation offers several advantages, including high efficiency, safety, and freedom from seasonal and geographical limitations. Microorganisms such as fungi, yeasts, and bacteria can be employed in the fermentation of GABA. Lactic acid bacteria, which are considered as Generally Recognized as Safe (GRAS) microorganisms, have been reported to be capable of producing GABA in large quantities. Strains such as *L. plantarum* (Park et al., 2021), *L. brevis* (Kanklai et al., 2021), *Pediococcus pentosus* (Thuy et al., 2021), *Lactobacillus fermentum* (Rayavarapu et al., 2019), and *Lactococcus lactis* (Santos-Espinosa et al., 2020) have been found to possess the ability to produce GABA. GABA-rich fermented foods have also been studied. For example, soy yogurt was fermented with *L. plantarum*. The results demonstrated its efficacy in restoring insulin-producing function in hyperglycemic mice and promoting the re-establishment of a healthy gut microbiota (Weng et al., 2023). Besides, co-culture strategies are also employed as significant methods to enhance fermentation quality and promote the accumulation of GABA. An illustrative instance involved the fermentation of turmeric and roasted soybean flour using the microorganism strains *Bacillus subtilis* HA and *L. plantarum* K154. In the final stage of fermentation, co-fermentation exhibited a notably higher GABA accumulation, surpassing the single-fermented system by as much as 1.78% (Lim et al., 2016).

Currently, the majority of research on GABA-enriched fermented foods has primarily centered on milk and vegetable juice (Maria Ramos and Maria Poveda, 2022; Jin et al., 2023; Weng et al., 2023). Limited attention has been directed toward fermented vegetables and this is particularly true for bamboo shoots. Meanwhile, reports on the synergistic fermentation of bamboo shoots, providing both quality and functionality, are quite scarce. In our study, *L. plantarum* R1 and *L. brevis* R2 were selected based on their commendable potential probiotic attributes and noteworthy GABA production, respectively. We endeavored to enhance the GABA content in fermented bamboo shoots through a mixed fermentation process involving these two strains. Concurrently, we aimed to achieve enhanced nutritional quality while ensuring the safety and desirable sensory attributes. This strategic approach might hold promise for the development of functional fermented bamboo shoots.

## 2 Materials and methods

### 2.1 Isolation of LAB strains

LAB strains were isolated from fermented bamboo shoots obtained from Liuzhou, Guangxi, China. The isolation process involved serial dilutions of 1 mL of fermented bamboo shoot liquid using sterile saline (0.9% NaCl, w/v). Then it was plated onto Chalmers (MC) agar medium (Hope Bio-Technology Co., Ltd., Qingdao, China). The plates were then incubated at 37°C for 48 h. After the incubation period, single colonies with transparent circle were selected and streaked onto de Man Rogosa Sharpe (MRS) agar medium (Hope Bio-Technology Co., Ltd., Qingdao, China) to obtain pure cultures. The isolated strains were initially identified using Gram staining and peroxidase tests. Subsequently, the pure cultures were preserved by storing them at −80°C with the addition of 40% glycerol for further analysis.

### 2.2 Molecular identification

DNA extraction from the isolates was extracted using the Bacteria DNA Isolation Mini Kit (Vazyme Biotech Co., Ltd., Nanjing, China). PCR reactions were carried out in a total volume of 50 μL, containing 25 μL of Mix (Vazyme Biotech Co., Ltd., Nanjing, China), 21 μL of sterilized ultrapure water, 2 μL of forward primer 27F (5’-AGAGTTTGATCCTGGCTCAG-3′), 2 μL of reverse primer 1492R (5’-GGTTACCTTGTTACGACTT-3′), and 5 μL of DNA template. The PCR amplification procedure was performed using a XP Thermal Cycler (Bioer Technology Co., Ltd., Hangzhou, China). The PCR conditions were as follows: an initial denaturation step of 95°C for 5 min, followed by 30 cycles of denaturation at 95°C for 30 s, annealing at 50°C for 30 s, and extension at 72°C for 90 s. Finally, there was a single extension step at 72°C for 10 min. The PCR product was purified and sequenced by Tsingke Biotech Co., Ltd., Beijing, China. BLAST analysis was conducted using the NCBI database.^1^

### 2.3 Potential probiotic characterization

#### 2.3.1 Acid and bile salts tolerances

The acid and bile salt tolerances of the LAB strains were performed as described by Xia et al. (2021) with slight modifications. To assess tolerance to acidic conditions, the MRS broth was adjusted to pH 2.5 and pH 3.5 using hydrochloric acid. Tolerance to bile salts was examined by supplementing the MRS broth with 0.3 and 0.5% (w/v) of bile salts (Wokai Biotechnology Co., Ltd., Beijing, China). The LAB cells cultured for 24 h were washed three times with sterile phosphate buffer saline (PBS) of pH 7.4 and resuspended in PBS. Subsequently, 100 μL of the bacterial suspension was mixed with 10 mL of modified MRS broth (MRS adjusted pH or supplemented with bile salts) and incubated for 3 h at 37°C. To determine the viable cells, a plate count method was employed using MRS agar, and the results were expressed as log colony forming unit (CFU) /mL. The survival rate was calculated using the following equation:


$$\mathrm{Survival}\;\mathrm{rate}\;\left(\%\right)=\left(\log\;N/\log\;N_{0}\right)\times 100$$

where Log N is the log number of viable cells at the end of the test and Log N_0_ is the log number of initial viable cells.

#### 2.3.2 Antimicrobial activity

The antimicrobial activity was measured using the agar well diffusion method (Abdollahzadeh et al., 2014). The LAB cells were incubated overnight and the supernatant was collected by centrifugation at 1,902 × g for 15 min. Indicator strains including *Escherichia coli* ATCC35218, *Salmonella enteritidis*, *Pseudomonas aeruginosa* ATCC27853, and *Staphylococcus aureus* ATCC6538 were selected as common pathogens. The overnight culture of indicator bacteria was spread evenly on LB agar (Hope Bio-Technology Co., Ltd., Qingdao, China). Then wells were prepared using a 0.8 cm Oxford cup and 150 μL of the supernatant was added. After incubating at 37°C for 12 h, the diameter of the inhibition zone was measured. Uninoculated MRS broth was used as a control in the experiment.

#### 2.3.3 Cell surface hydrophobicity

The determination of cell surface hydrophobicity was conducted following the method described by Albayrak and Duran (2021). Briefly, the LAB cells were suspended in PBS to obtain an OD_600_ of 0.8 (A_0_). Subsequently, 3 mL of the cell suspension was added to 1 mL of xylene and ethyl acetate, respectively, vortexed for 2 min, and allowed to stand at 37°C for 1 h. The absorbance (A_t_) of the aqueous phase at 600 nm was determined. Cell surface hydrophobicity (%) was calculated according to the following formula:


$$\mathrm{Cell}\;\mathrm{surface}\;\mathrm{hydrophobicity}\;\left(\%\right)=\left(1-A_{t}/A_{0}\right)\times 100$$

#### 2.3.4 Auto-aggregation assay

The auto-aggregation assay of the LAB cells was performed based on the method previously described (Albayrak and Duran, 2021) with slight modifications. The cell suspensions were adjusted to approximately an OD_600_ of 0.8 (A_0_). Subsequently, a total of 3 mL of the cell suspensions were transferred to a test tube, vortexed for 10 s and kept at 37°C for 6 h. The absorbance (A_t_) of the upper liquid layer at 600 nm was determined at 2 and 6 h, respectively. Auto-aggregation (%) was calculated according to the following formula:


$$\mathrm{Auto}-\mathrm{aggregation}\;\left(\%\right)=\left(1-A_{t}/A_{0}\right)\times 100$$

#### 2.3.5 Antibiotic susceptibility

The antibiotic susceptibility testing was performed using the Kirby Bauer Disk diffusion method (García-Núñez et al., 2022). The strains were cultured in MRS broth to a concentration of 10^8^ CFU/mL and spread evenly on the MRS agar plates. Antibiotic disks (Oxoid Ltd., Basingstoke, Hans, United Kingdom) containing various antibiotics, including Amikacin (30 μg), Chloramphenicol (30 μg), Kanamycin (30 μg), Sulfamethoxazole/Trimethoprim (25 μg), Ampicillin/Sulbactam (20 μg), Tetracycline (30 μg), Cefotaxime (30 μg), and Nalidixic Acid (30 μg) were placed on the agar surface. The plates were then incubated at 37°C for 24 h. The diameter of the inhibition zone was measured, and the results were interpreted as follows: resistant (≤ 15 mm), intermediate (15–21 mm), or susceptible (≥ 21 mm).

### 2.4 GABA measurement

LAB strains were cultured in MRS broth supplemented with 1% (w/v) monosodium glutamate (MSG) for 24 h. Then the culture supernatant was obtained through centrifugation at 6,010 × g for 5 min. To conduct the initial screening of GABA-producing strains, thin-layer chromatography (TLC) was employed (Park et al., 2021). A developing solvent composed of butyl alcohol, glacial acetic acid, and distilled water in a ratio of 4:1:1 (v:v:v) was used. Visualization of the TLC plates was carried out using a 0.5% (w/v) ninhydrin solution.

Quantitative analysis of GABA was conducted using an ultra-performance liquid chromatography (UPLC) method (Kim et al., 2022). An Acquity UPLC^®^ system (Waters Corporation Inc., Milford, United States) coupled with a 2,996 photodiode array detector was employed. The supernatant obtained from the LAB strains was derivatized with o-phthalaldehyde (OPA) (Macklin, Biochemical Co., Ltd., Shanghai, China) for 2 min, followed by filtration using a 0.45 μm filter. The analysis of GABA content was performed using an InfinityLab Poroshell 120 EC-C18 column (3.0 × 150 mm, 2.7 μm) (Agilent Technologies Inc., Santa Clara, United States). The mobile phase solution system was comprised of (A) 5 g/L of sodium acetate at pH 7.2 and (B) a mixture of acetonitrile, methanol, and sodium acetate in a ratio of 40:40:20 (v:v:v) at pH 7.2. The gradient elution conditions of mobile phase A were applied as following: 0 min, 92%; 12 min, 50%; 15 min, 50%; 18 min, 92%. The system was operated at a flow rate of 0.8 mL/min at 30°C with detection performed at a wavelength of 340 nm.

### 2.5 Bamboo shoots fermentation

#### 2.5.1 Sample preparation

*L. plantarum* R1 and *L. brevis* R2 were suspended with PBS for use as fermentation agents. Fresh bamboo shoots were sourced from Guangdong, China. After cleaning, old roots were removed, and the shoots were peeled and cut into uniform strips. The bamboo shoots were placed in sterilized 200 mL glass jars at a ratio of 1:2 (w:v) with cooled boiling water. *L. plantarum* R1, *L. brevis* R2, mixture of *L. plantarum* R1 and *L. brevis* R2 (1:1) were inoculated with 3% (v/v) of the total inoculum, respectively. The natural fermentation group did not receive any LAB inoculation. The glass jars were then sealed and the fermentation was conducted at room temperature (26°C) for a duration of 14 days.

#### 2.5.2 Determination of pH value and total titratable acidity

During the fermentation process, samples were collected every 2 days for pH and TTA analysis. The bamboo shoots (5 g) were homogenized with 10 mL of water and centrifuged at 6,010 × g for 5 min. The pH of the supernatant was measured using a pH meter (INESA Co., Ltd., Shanghai, China). The determination of TTA was conducted as described by Zhao et al. (2022) and the TTA was expressed as g/100 g with lactic acid as a standard.

#### 2.5.3 Determination of nitrite content

For nitrite analysis, the bamboo shoots (5 g) were used to precipitate proteins with a combination of potassium ferricyanide and zinc acetate. Afterward, it was set aside to allow for the removal of fat. The resulting supernatant, obtained after filtration, was employed to determine nitrite content using the N-(1-naphthyl)-ethylenediamine dihydrochloride spectrophotometric method according to GB 5009.33–2016 in China.

#### 2.5.4 Determination of GABA content

After 14 days of fermentation, 5 g of the bamboo shoots were mixed with 10 mL of water, subjected to ultrasound to promote dissolution, and then filtered through a 0.22 μm filter membrane to collect the filtrate. The filtrate was further derivatized using OPA and the content of GABA was determined using the UPLC method.

#### 2.5.5 Determination of nutritional composition

Following a 14-day fermentation period, the protein, total sugars, reducing sugars, fat, and crude fiber contents in bamboo shoots were assessed. Protein content was determined using Bradford reagent (Sangon Biotech Co., Ltd., Shanghai, China). The anthrone method was employed for the quantification of total sugars, while reducing sugars were assessed using the dinitrosalicylic acid method (Singhal et al., 2021). The determination of fat content adhered to the methodology outlined by Hellwig et al. (2022). The evaluation of crude fiber content was measured subsequent to acid and alkali treatment, following the procedure described by Azeez et al. (2022).

#### 2.5.6 Sensory analysis

After a 14-day fermentation period, the bamboo shoots were selected for sensory evaluation. A total of 5 descriptors containing color, flavor, taste, texture, and overall acceptability were selected. The blind evaluation panel comprised 10 judges (5 men and 5 women) experienced in sensory evaluation. Scores were assigned on a scale ranging from 1 (dislike extremely) to 10 (like extremely).

### 2.6 Statistical analysis

Principal component analysis (PCA) and heat map were performed using the combination of SPSS 26.0 (IBM Inc., United States) and OriginPro 2023b (OriginLab Corporation, Northampton, United States). Data were normalized before applying the feature selection algorithms and weighting results presented between 0 and 1, in which weight closer to 1 indicated the priority of the studied strains. All experiments were repeated three times and all data were expressed as means ± standard deviation. The statistical analysis was completed using SPSS 26.0 (IBM Inc., United States) and significant differences between groups were evaluated by the Duncan test. The statistical significance level was defined as *p* < 0.05.

## 3 Result and discussion

### 3.1 Isolation and molecular identification of LAB strains

Insoluble calcium carbonate in MC medium can be converted to calcium lactate by lactic acid produced by LAB strains, resulting in the appearance of transparent circles. Thirty-five strains that could produce large transparent circles were isolated, all of which were Gram-positive, catalase-negative, and rod-shaped or globular. Based on the similar phenotypes, 20 isolates were selected for 16S rRNA gene identification and the sequences were analyzed using BLAST. As shown in Table 1, the isolates were identified as *Lactiplantibacillus plantarum*, *Levilactobacillus brevis*, *Pediococcus acidilactici*, *Pediococcus pento-saceus*, *Limosilactobacillus fermentum*, and *Lacticaseibacillus paracasei*. Sequences have been submitted to the NCBI database.

**Table 1:** Molecular identification of LAB strains by 16S rRNA gene sequence.

Strains	Nucleotide similarity	Identification results	Accession number
R1	99.66%	*Lactiplantibacillus plantarum*	OR481916
R2	99.86%	*Levilactobacillus brevis*	OR481917
R5	99.38%	*Levilactobacillus brevis*	OR481918
R6	99.59%	*Lactiplantibacillus plantarum*	OR481919
R7	99.45%	*Levilactobacillus brevis*	OR481920
R8	99.52%	*Levilactobacillus brevis*	OR481921
R9	99.12%	*Pediococcus acidilactici*	OR481922
R10	99.65%	*Pediococcus pentosaceus*	OR481923
R12	96.08%	*Lactiplantibacillus plantarum*	OR481924
R14	99.79%	*Lactiplantibacillus plantarum*	OR481925
R17	99.79%	*Lactiplantibacillus plantarum*	OR481926
R19	99.79%	*Lactiplantibacillus plantarum*	OR481927
R22	99.24%	*Levilactobacillus brevis*	OR481928
R23	99.79%	*Lactiplantibacillus plantarum*	OR481929
R25	100.00%	*Lactiplantibacillus plantarum*	OR481930
L1	99.59%	*Lactiplantibacillus plantarum*	OR481931
C2	98.85%	*Limosilactobacillus fermentum*	OR481932
C3	99.53%	*Limosilactobacillus fermentum*	OR481933
C16	99.33%	*Limosilactobacillus fermentum*	OR481934
G1	97.95%	*Lacticaseibacillus paracasei*	OR481935

### 3.2 Potential probiotic properties

#### 3.2.1 Acid and bile salts tolerance

**Figure 1:**
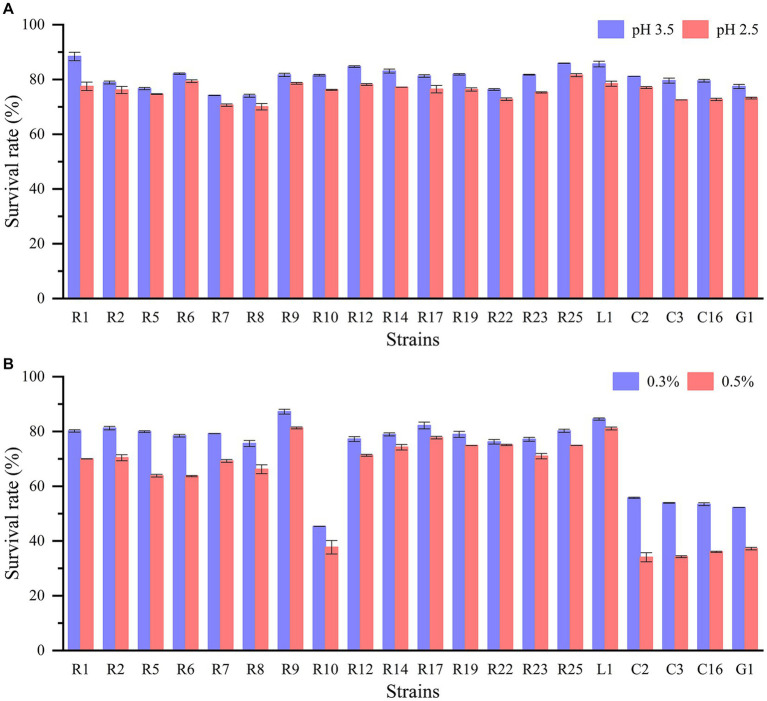
Acid and bile salt tolerance of LAB strains: **(A)** survival rate in pH 3.5 and pH 2.5; **(B)** survival rate in 0.3 and 0.5% bile salt.

The journey of lactic acid bacteria through the gastrointestinal tract confronts a series of survival challenges, including exposure to gastric acid, digestive enzymes, and bile salts. The capacity to withstand low pH levels and bile salts is especially imperative for the bacteria. All LAB strains displayed remarkable acid tolerance (Figure 1A). When cultured at pH 2.5 and pH 3.5 for 3 h, all strains demonstrated robust acid tolerance, with survival rates surpassing 70%. Additionally, the cell densities of all strains remained within the range of 10^6^–10^8^ CFU/mL. For example, *L. plantarum* R1 and *L. brevis* R2 achieved survival rates of 77.49 and 76.17%, respectively, at pH 2.5. On the other hand, there were significant differences in the tolerance of LAB isolates to bile salts (*p* < 0.05) (Figure 1B). *P. pentosaceus*, *L. fermentum*, and *L. paracasei* exhibited poor bile salts tolerance, with survival rates at approximately 50% when exposed to 0.3% bile salts. In contrast, other strains displayed higher bile salts tolerance, with *P. acidilactici* R9 and *L. plantarum* L1 demonstrating the highest bile salts tolerance, as their survival rates remained above 80% even in the presence of 0.5% bile salts. And other strains such as *L. plantarum* R1 and *L. brevis* R2 had greater than 70% survival in 0.5% bile salts. Indeed, extensive studies have consistently demonstrated that the tolerance of LAB to acid and bile salts varies significantly based on the species and specific strains being investigated. Experiments involving the exposure of LAB to acid and bile salts have demonstrated that some strains exhibited survival rates of up to 80% or higher. Conversely, there were strains that displayed limited survivability under these conditions (Tokatlı et al., 2015; Wang et al., 2021; Khushboo et al., 2023).

#### 3.2.2 Antimicrobial activity

One of the notable advantages of LAB is their capacity to inhibit the growth of foodborne pathogens. This inhibitory effect arises primarily from the production of various bioactive compounds, including organic acids, bacteriocins, and hydrogen peroxide (Chen J. et al., 2022). As shown in Table 2, the antimicrobial ability of the strains showed significant differences (*p* < 0.05). *L. plantarum* exhibited the most pronounced inhibitory effect against all four pathogens. *P. acidilactici* R9 and *P. pentosaceus* R10 also exhibited strong inhibitory abilities, with their inhibitory effects being more prominent against Gram-negative bacteria compared to Gram-positive bacteria. However, *L. fermentum* and *L. paracasei* displayed the weakest inhibitory abilities, particularly against Gram-positive bacteria, which is similar to the findings of Trindade et al. (2022) and Younas et al. (2022). This is seemingly attributed to the ability of LAB in inhibiting or eradicating biofilms (Shokri et al., 2018; Ibrahim et al., 2021), a mechanism that proves more challenging when confronted with the thicker peptidoglycan cell wall structure characteristic of Gram-positive bacteria.

**Table 2:** Inhibition circle (mm) against pathogens by LAB strains.

Species	Strains	*Staphylococcus aureus*	*Escherichia coli*	*Salmonella*	*Pseudomonas aeruginosa*
*L. plantarum*	R1	9.56 ± 0.14^bcd^	10.13 ± 1.12^a^	12.3 ± 0.72^b^	12.43 ± 0.45^bc^
	R6	9.63 ± 0.64^bcd^	9 ± 0.34^a^	12.59 ± 0.47^b^	11.89 ± 0.19^c^
	R12	8.54 ± 0.22^de^	9.42 ± 0.44^a^	13.49 ± 0.11^ab^	11.56 ± 0.41^c^
	R14	9.19 ± 0.5^bcde^	9.01 ± 0.28^a^	13.91 ± 0.36^a^	12.16 ± 0.31^c^
	R17	11.13 ± 0.05^a^	10 ± 0.95^a^	12.09 ± 0.5^b^	12.22 ± 0.89^bc^
	R19	9.62 ± 0.34^bcd^	9.39 ± 0.51^a^	12.54 ± 0.22^b^	13.19 ± 0.54^ab^
	R23	10.19 ± 0.16^ab^	9.88 ± 0.83^a^	12.48 ± 0.1^b^	11.97 ± 0.09^c^
	R25	8.98 ± 0.04^cde^	9.29 ± 0.44^a^	14.55 ± 1.07^a^	11.52 ± 0.42^c^
	L1	10.07 ± 0.85^bc^	10.26 ± 1.18^a^	14.04 ± 0.99^a^	13.69 ± 0.61^a^
*L. brevis*	R2	8.2 ± 0.42^e^	5.52 ± 1.82^bc^	7.65 ± 0.9^de^	7.82 ± 0.25^e^
	R5	0^g^	6.08 ± 1.75^b^	8.94 ± 0.52^d^	7.72 ± 0.48^e^
	R7	0^g^	3.71 ± 0.04^cd^	7.67 ± 0.32^de^	7.51 ± 0.42^e^
	R8	0^g^	4.77 ± 1.16^bcd^	8.76 ± 0.49^d^	7.32 ± 0.49^e^
	R22	8.37 ± 0.49^e^	3.25 ± 1.37^d^	6.74 ± 0.21^e^	6.98 ± 0.34^e^
*P. acidilactici*	R9	8.22 ± 0.33^e^	8.74 ± 0.7^a^	12.52 ± 0.44^b^	10.28 ± 0.58^d^
*P. pentosaceus*	R10	8.73 ± 0.73^de^	9.05 ± 0.24^a^	12.41 ± 0.09^b^	12.1 ± 0.58^c^
*L. fermentum*	C2	5.85 ± 0.78^f^	5.53 ± 1.06^bc^	8.86 ± 0.88^d^	7.82 ± 0.57^e^
	C3	6.46 ± 0.5^f^	4.26 ± 0.53^bcd^	9.9 ± 0.68^cd^	7.89 ± 0.57^e^
	C16	6.13 ± 0.25^f^	4.14 ± 0.07^bcd^	10.6 ± 1.02^c^	7.27 ± 0.2^e^
*L. paracasei*	G1	0^g^	2.01 ± 0.8^e^	4 ± 0.23^f^	3.27 ± 0.31^f^

The results were determined by subtracting the diameter of the Oxford cup from the diameter of the transparent ring and expressed as mean ± standard deviation (*n* = 3). Means in the same column with different superscript letters indicate significant differences among strains (*p* < 0.05).

#### 3.2.3 Cell surface hydrophobicity and auto-aggregation

**Figure 2:**
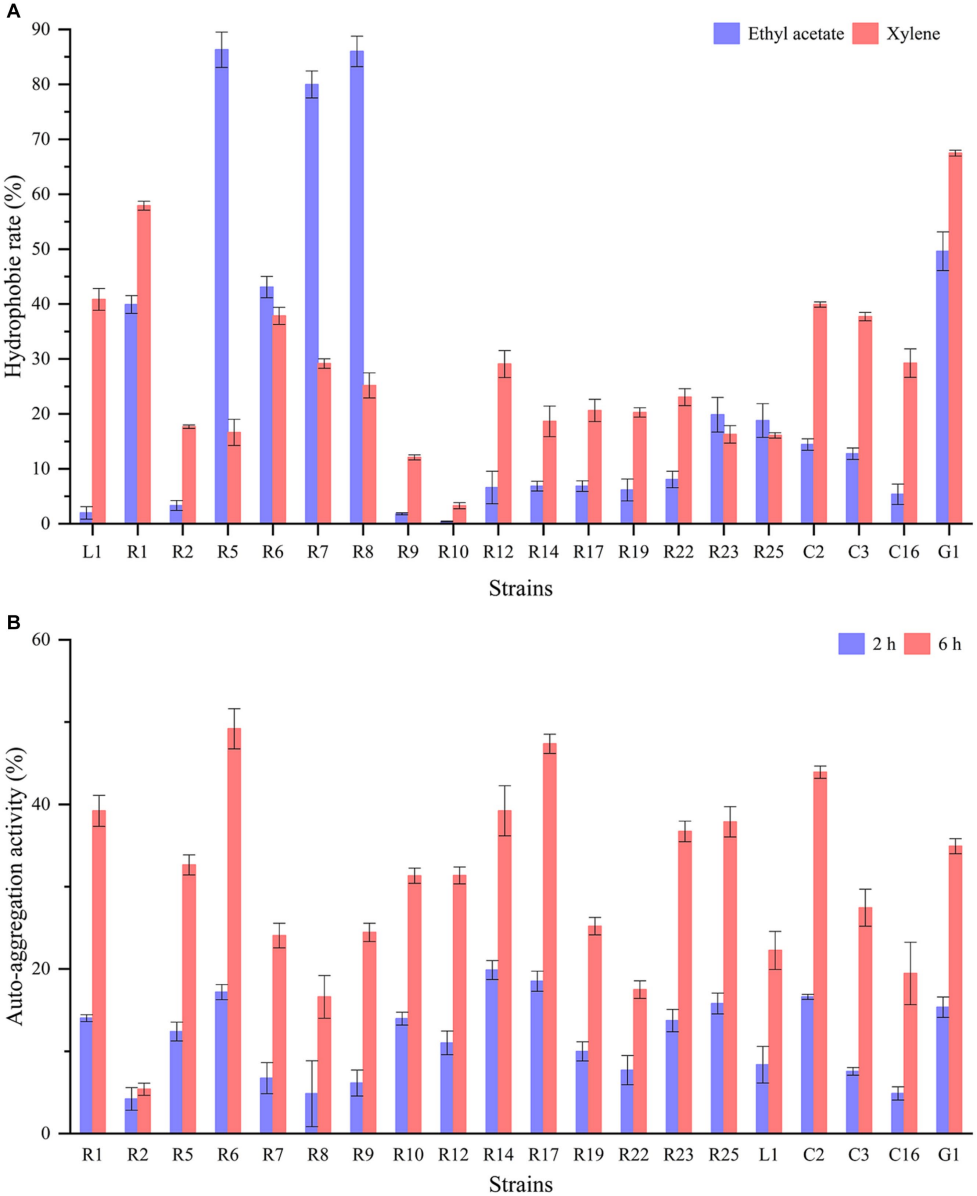
Hydrophobicity and auto-aggregation of LAB strains: **(A)** hydrophobicity in ethyl acetate and xylene; **(B)** auto-aggregation after 2 h and 6 h.

The cell surface hydrophobicity and auto-aggregation ability of LAB strains are closely linked to their capacity to adhere to epithelial cells and carry out their functional roles. Significant differences (p < 0.05) in cell surface hydrophobicity were observed among different strains (Figure 2A). *L. paracasei* G1 displayed relatively high hydrophobicity in both ethyl acetate and xylene, exceeding 50%. Following closely were *L. plantarum* R1 and R6, both exhibiting hydrophobicity levels surpassing 40%. Notably, *L. brevis* R5, R7, and R8 showed substantial disparities with hydrophobicity exceeding 80% in ethyl acetate but falling below 30% in xylene. In contrast, *P. acidilactici* R9 and *P. pentosaceus* R10 displayed minimal hydrophobicity. Figure 2B illustrates the auto-aggregation ability of the isolates. The rate of auto-aggregation increased over time for all strains. Among the strains tested, *L. plantarum* R6 exhibited the most notable auto-aggregation ability, achieving a rate of 49.18% after a 6-h standing period. In stark contrast, *L. brevis* R2 demonstrated the lowest auto-aggregation ability, registering only 5.39% after 6 h. Wang et al. (2021) reported that the hydrophobicity in xylene and auto-aggregation of the commercial strain *L. rhamnosus* GG were 14.35 and 20.35%, respectively. In another study, the hydrophobicity in xylene of LAB strains isolated from artisanal white cheese ranged from 1 to 41.6%. And hydrophobicity in ethyl acetate showed a broader range from 8.2 to 50.2%. Interestingly, there was no significant correlation between these two solvents (Albayrak and Duran, 2021). It has been demonstrated that the adhesion properties of LAB are typically strain-specific rather than species-specific, and are subject to influence by factors such as growth conditions, incubation time, and bacterial surface structure (García-Cayuela et al., 2014).

#### 3.2.4 Antibiotic susceptibility

The emergence of microbial resistance, attributed to the widespread use of antibiotics, poses a significant threat in various fields including medicine and food production. The antibiotic susceptibility of the isolates is presented in Table 3. It is noteworthy that all strains demonstrated either no susceptibility or poor susceptibility to antibiotics including Kanamycin, Nalidixic Acid, and Amikacin. Furthermore, all strains excluding *L. plantarum* also exhibited no susceptibility to Sulfamethoxazole/Trimethoprim. In contrast, Tetracycline, Chloramphenicol, Ampicillin/Sulbactam, and Cefotaxime were found to be effective in inhibiting the growth of all strains. Same as our findings, studies have found that LAB strains possess resistance to antibiotics such as Nalidixic Acid, Kanamycin, Amikacin, Gentamicin, and Streptomycin (García-Núñez et al., 2022; Duche et al., 2023). Fortunately, this resistance is considered as an inherent characteristic of LAB. Researchers have confirmed that some resistance is intrinsic to LAB, as resistance genes are encoded within their chromosomes and do not undergo horizontal transmission (Argyri et al., 2013). There are also instances where LAB exhibit apparent resistance despite lacking the associated resistance genes (Ou et al., 2022). Additionally, it has been assumed that membrane impermeability may also contribute to the resistance(Elkins and Mullis, 2004). Conversely, numerous studies have noted that LAB strains display sensitivity to Tetracycline, Chloramphenicol, and Cefotaxime (Albayrak and Duran, 2021; García-Núñez et al., 2022; Ou et al., 2022; Duche et al., 2023), with variations in susceptibility observed among different strains and species toward the same antibiotic.

**Table 3:** Antibiotic susceptibility of LAB strains.

Species	Strains	AK	C	K	SXT	SAM	TE	CTX	NA
*L. plantarum*	R1	14(R)	30(S)	0(R)	17(I)	32(S)	21(S)	37(S)	0(R)
	R6	10(R)	26(S)	0(R)	18(I)	26(S)	21(S)	27(S)	0(R)
	R12	10(R)	30(S)	0(R)	21(S)	30(S)	22(S)	30(S)	0(R)
	R14	13(R)	29(S)	0(R)	22(S)	31(S)	20(I)	27(S)	0(R)
	R17	13(R)	29(S)	0(R)	23(S)	29(S)	21(S)	29(S)	0(R)
	R19	9(R)	28(S)	0(R)	18(I)	32(S)	20(I)	32(S)	0(R)
	R23	12(R)	30(S)	0(R)	20(I)	29(S)	23(S)	29(S)	0(R)
	R25	10(R)	26(S)	0(R)	17(I)	27(S)	19(I)	21(S)	0(R)
	L1	12(R)	29(S)	0(R)	23(S)	31(S)	22(S)	32(S)	0(R)
*L. brevis*	R2	0(R)	24(S)	0(R)	0(R)	16(I)	15(I)	19(I)	0(R)
	R5	14(R)	32(S)	0(R)	0(R)	26(S)	23(S)	23(S)	0(R)
	R7	12(R)	34(S)	0(R)	0(R)	26(S)	25(S)	28(S)	0(R)
	R8	16(I)	34(S)	0(R)	0(R)	25(S)	27(S)	27(S)	0(R)
	R22	0(R)	31(S)	0(R)	0(R)	18(I)	20(I)	21(S)	0(R)
*P. acidilactici*	R9	0(R)	26(S)	0(R)	0(R)	18(I)	18(I)	21(S)	0(R)
*P. pentosaceus*	R10	0(R)	28(S)	0(R)	0(R)	19(I)	21(S)	25(S)	0(R)
*L. fermentum*	C2	11(R)	26(S)	0(R)	0(R)	30(S)	23(S)	23(S)	0(R)
	C3	12(R)	26(S)	0(R)	0(R)	29(S)	22(S)	24(S)	0(R)
	C16	12(R)	27(S)	0(R)	0(R)	30(S)	25(S)	25(S)	0(R)
*L. paracasei*	G1	12(R)	27(S)	0(R)	0(R)	33(S)	24(S)	26(S)	0(R)

The diameters of the transparent rings (mm) are expressed as an integer. AK, Amikacin (30 μg); C, Chloramphenicol (30 μg); Kanamycin (30 μg); SXT, Sulfamethoxazole/Trimethoprim (25 μg); SAM, Ampicillin/Sulbactam (20 μg); TE, Tetracycline (30 μg); CTX, Cefotaxime (30 μg); NA, Nalidixic Acid (30 μg). R, resistant (≤15 mm); I, intermediate (15–21 mm); S, susceptible (≥21 mm).

#### 3.2.5 Selection of LAB strains with favorable potential probiotic properties

**Figure 3:**
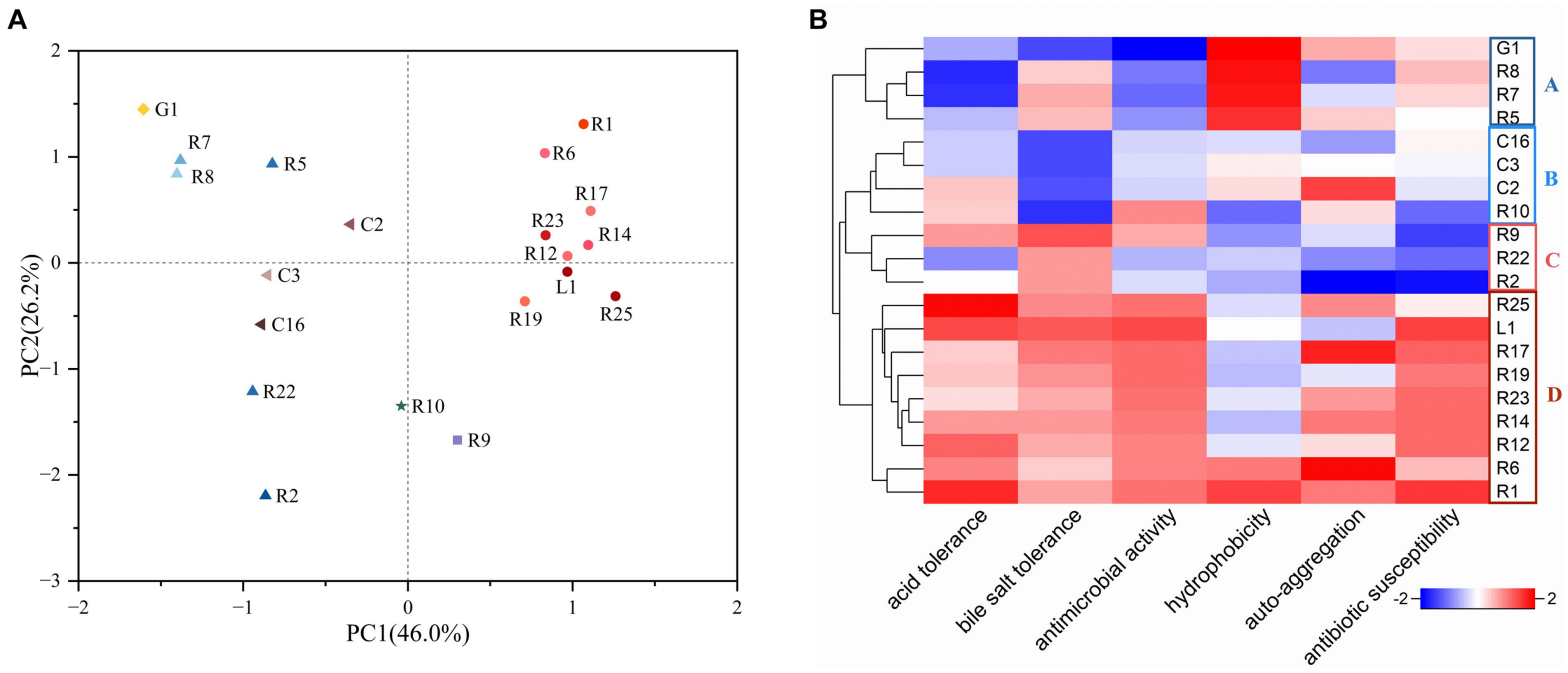
Selection of LAB strains with favorable probiotic properties (acid and bile salt tolerance, antimicrobial activity, hydrophobicity, auto-aggregation, and antibiotic susceptibility). **(A)** Principal component analysis (PCA), the percentages: the values contributed by the principal components to the sample differences; **(B)** Heat map, the redder the color is, the better the probiotic property is.

PCA and heat map have emerged as pivotal tools for the screening of functional strains (Wang et al., 2021). To comprehensively assess and compare the properties of the isolates for the selection of probiotic candidates for bamboo shoot fermentation, we employed PCA, considering six attributes: acid resistance, bile salt resistance, antimicrobial activity, cell surface hydrophobicity, auto-aggregation, and antibiotic susceptibility. The same attribute with multiple results was compared using the comprehensive rating method with equal weight assigned to each result. As shown in Figure 3A, principal component 1 (PC1) and principal component 2 (PC2) occupied 46.0 and 26.2% of the variance, respectively. The distribution of the strains was observed across four quadrants. *L. plantarum* were densely concentrated in quadrants one and four, whereas *L. fermentum*, *L. brevis*, and *L. paracasei* were primarily distributed in quadrants two and three. Additionally, a heat map was utilized to visualize the properties of LAB strains (Figure 3B). The strains were categorized into four groups: A, B, C and D. Group A exhibited notable cell hydrophobicity but limited acid tolerance and antimicrobial activity, while group B displayed low bile salt tolerance. Group D excelled in all other characteristics, except for hydrophobicity. Intriguingly, all members of Group D were identified as *L. plantarum*. Based on the combined insights from PCA and the heatmap, we concluded that *L. plantarum* R1 showcased the most potential probiotic properties among the tested strains. Consequently, *L. plantarum* R1 was selected as one of the strains for subsequent bamboo shoot fermentation.

### 3.3 Screening of LAB strains for high GABA production

**Figure 4:**
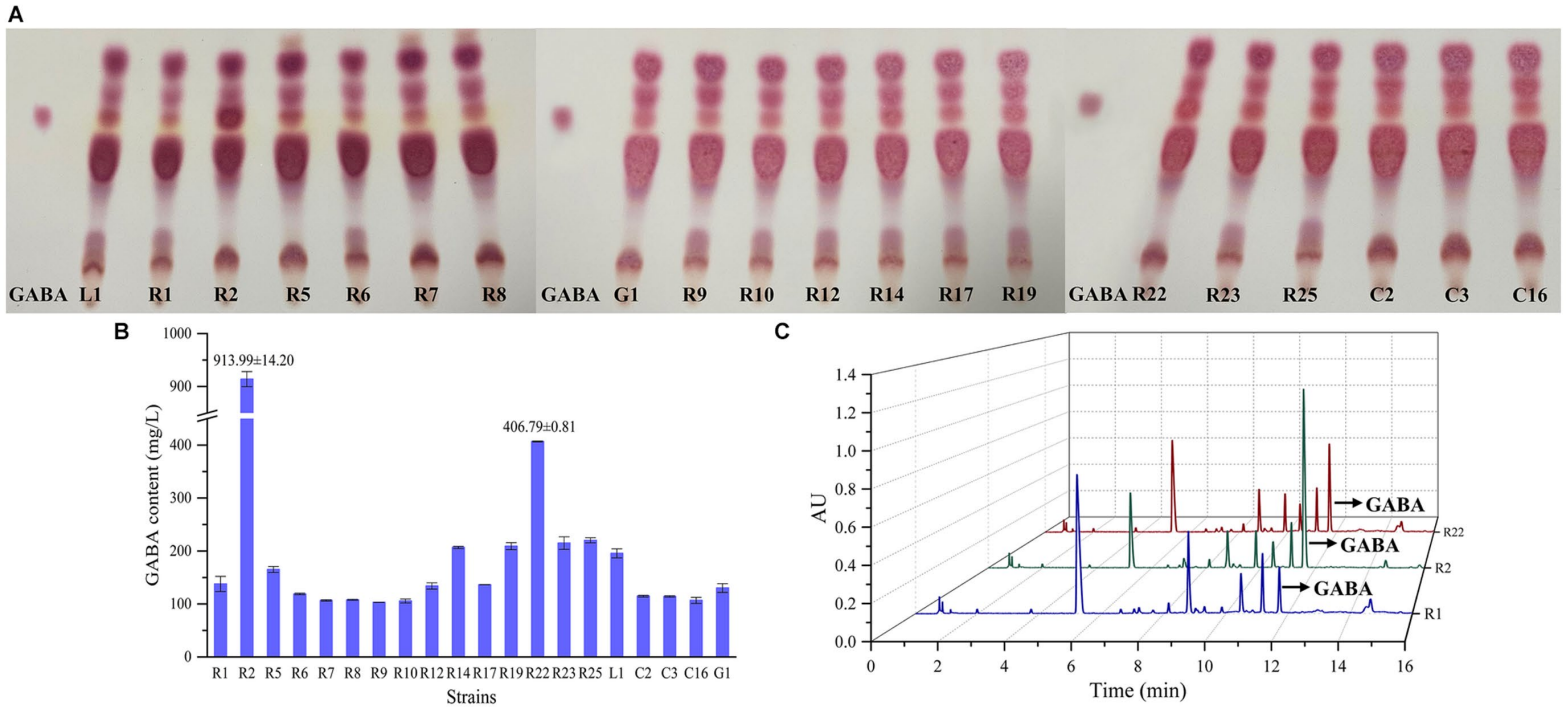
Screening of GABA-producing LAB strains: **(A)** thin layer chromatography (TLC) screening of GABA–producing LAB; **(B)** GABA content (μg/mL) of LAB using ultra-performance liquid chromatography (UPLC) method; **(C)** chromatographic profile of GABA content in supernatants of *L. plantarum* R1, *L. brevis* R2 and *L. brevis* R22 analyzed, the retention time of GABA is about 11.7 min.

The preliminary screening of GABA-producing strains was conducted through TLC (Figure 4A). All strains displayed spots that closely matched the position of the GABA standard. Notably, the spot corresponding to *L. brevis* R2 exhibited the most pronounced coloration, followed by *L. brevis* R22. Following the initial screening, a quantitative assessment of GABA production was carried out using UPLC method, as depicted in Figures 4B,C. *L. brevis* R2 emerged as the most prolific GABA producer, showcasing a significantly high GABA production level (*p* < 0.01) of 913.99 ± 14.2 mg/L. Furthermore, *L. brevis* R22 also demonstrated a noteworthy distinction in GABA production compared to the other strains, with a recorded production level of 406.79 ± 0.81 mg/L. Consequently, *L. brevis* R2 was selected as another strain for subsequent bamboo shoot fermentation.

GABA-producing LAB have been extensively screened from a variety of fermented foods due to their remarkable ability to convert L-glutamic acid into GABA. For instance, 262.06 ± 15.42 mg/L GABA was successfully produced by *L. brevis* DSM 32386 isolated from traditional “wild” Alpine cheese (Mancini et al., 2019). In another study, *L. plantarum* FNCC 260 isolated from fermented cassava was found to produce 809.2 mg/L of GABA after 60 h of culture (Yogeswara et al., 2021). Furthermore, several strains including *L. fermentum*, *P. pentosaceus*, and *L. brevis*, identified from various fermented foods, have shown the capability to produce GABA at high yields (Rayavarapu et al., 2019; Pakdeeto et al., 2022). It has been noted that all LAB strains possessing the GAD gene exhibit the capacity to synthesize GABA (Pannerchelvan et al., 2023). Additionally, the yield of GABA is influenced by various factors such as concentration of L-glutamic acid, pH, temperature, and the size of inoculum.

### 3.4 Bamboo shoots fermentation

#### 3.4.1 Changes in pH and TTA during bamboo shoots fermentation

**Figure 5:**
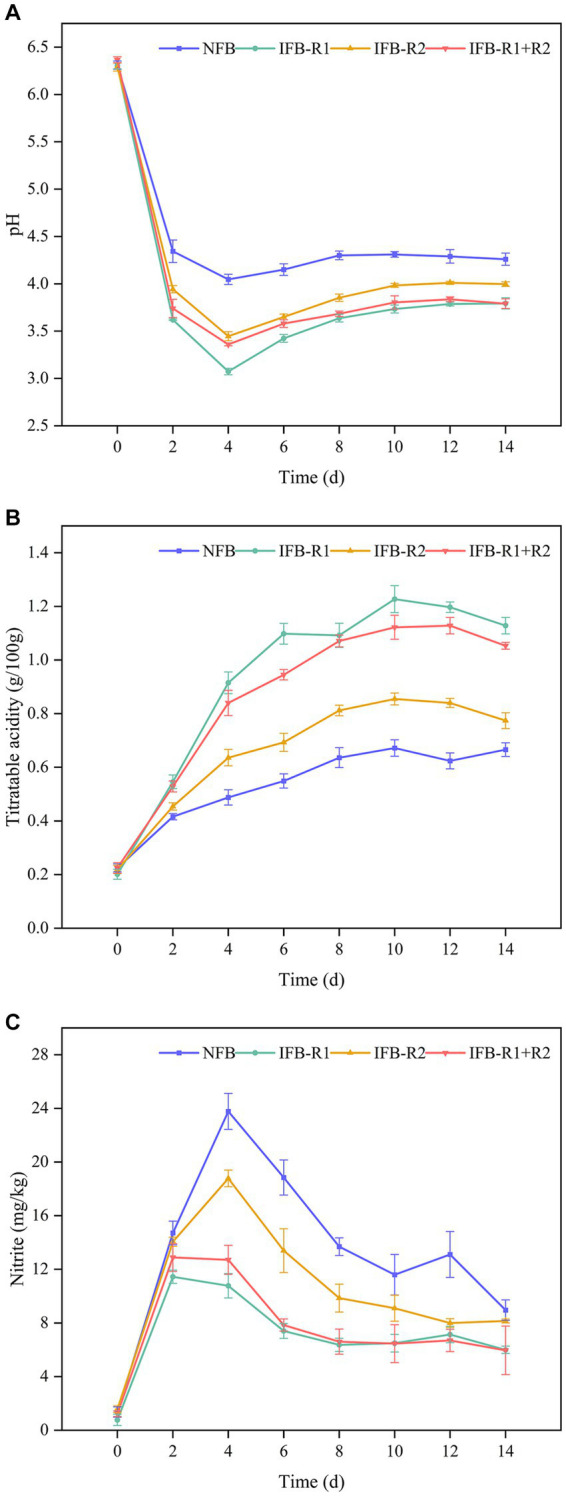
pH, total titratable acidity (TTA) and nitrite content of bamboo shoots during fermentation process: **(A)** pH values; **(B)** TTA; **(C)** nitrite content. NFB: naturally fermented bamboo shoots; IFB-R1: fermented bamboo shoots inoculated with *L. plantarum* R1; IFB-R2: fermented bamboo shoots inoculated with *L. brevis* R2; IFB-R1 + R2: fermented bamboo shoots inoculated with *L. plantarum* R1 and *L. brevis* R2.

In fermented foods, pH and TTA plays a crucial role in determining taste and serves as a key indicator of maturity. As shown in Figure 5A, the pH levels in all groups experienced a rapid decline in the initial 2 days. Notably, the pH in the inoculated fermentation was lower than that in the natural fermentation, with *L. plantarum* R1 exhibiting the most pronounced effect on pH reduction. By the 4th day, pH levels began to increase and gradually stabilized within the range of 3.7–4.3. At the end of fermentation, the pH values were 4.26 for naturally fermented bamboo shoots (NFB), 3.79 for inoculated *L. plantarum* R1 (IFB-R1), 4.00 for inoculated *L. brevis* R2 (IFB-R2), and 3.79 for synergistic fermentation by both strains (IFB-R1 + R2). The TTA changes shown in Figure 5B revealed a trend of rapid increase followed by a gradual leveling off in all groups. Inoculation with *L. plantarum* R1 resulted in the most significant increase in TTA, reaching a peak value of 1.23 g/100 g on the 10th day of fermentation, which was 1.8 times higher than that of natural fermentation. This was followed by the synergistic use of *L. plantarum* R1 and *L. brevis* R2 with a peak value of 1.11 g/100 g. Our findings demonstrate that inoculated fermentation led to a more substantial reduction in pH and increase in TTA, effectively shortening the fermentation cycle compared to natural fermentation. This aligns with prior studies (Zhang et al., 2022; Chen et al., 2023). The introduction of LAB strains swiftly established dominance in the fermentation process. Their growth and metabolic activity resulted in the production of substantial acidic compounds, consequently lowering the pH values and inhibiting the growth of pathogenic microorganisms throughout the fermentation process. In addition, *L. plantarum* R1 exhibited higher acid production capability compared to *L. brevis* R2. This is attributed to the fact that *L. plantarum* R1, a homofermentative strain, solely converted glucose to lactic acid during fermentation. On the other hand, the heterofermentative strain, *L. brevis* R2, produced additional products such as carbon dioxide and ethanol alongside lactic acid.

#### 3.4.2 Changes in nitrite during bamboo shoots fermentation

Excessive intake of nitrite can lead to methemoglobinemia and elevate the risk of gastrointestinal cancers, underscoring the critical need to control nitrite levels in fermented bamboo shoots. As illustrated in Figure 5C, in the initial stages of fermentation, nitrite levels exhibited a gradual increase, with the addition of LAB strains demonstrating a mitigating effect, notably more pronounced with the use of *L. plantarum* R1. Subsequently, nitrite levels decreased, reaching 8.95 ± 0.76 mg/kg (NFB), 6.00 ± 0.27 mg/kg (IFB-R1), 8.15 ± 0.15 mg/kg (IFB-R2), and 5.96 ± 1.81 mg/kg (IFB-R1 + R2) at the end of fermentation. It is evident that the addition of *L. plantarum* R1 proved more effective in controlling nitrite levels, whether in mono-fermentation or co-fermentation. Fortunately, all four sets of data complied with the Chinese regulatory limits for nitrite content in fermented vegetables (< 20 mg/kg, GB 2762–2022). Consistent with our findings, studies by Wu R. et al. (2015) and Yu et al. (2023) also observed a pattern of initial nitrite increase followed by a decline during vegetable fermentation. This phenomenon might be attributed to the activity of nitrate-reducing microorganisms capable of converting nitrate to nitrite under anaerobic conditions in the early stages of fermentation. However, as fermentation progresses, LAB assumed dominance as the primary flora, which created an acidic environment that not only inhibited the growth of nitrate-reducing bacteria but also contributed to nitrite metabolism.

#### 3.4.3 Changes in GABA during bamboo shoots fermentation

**Figure 6:**
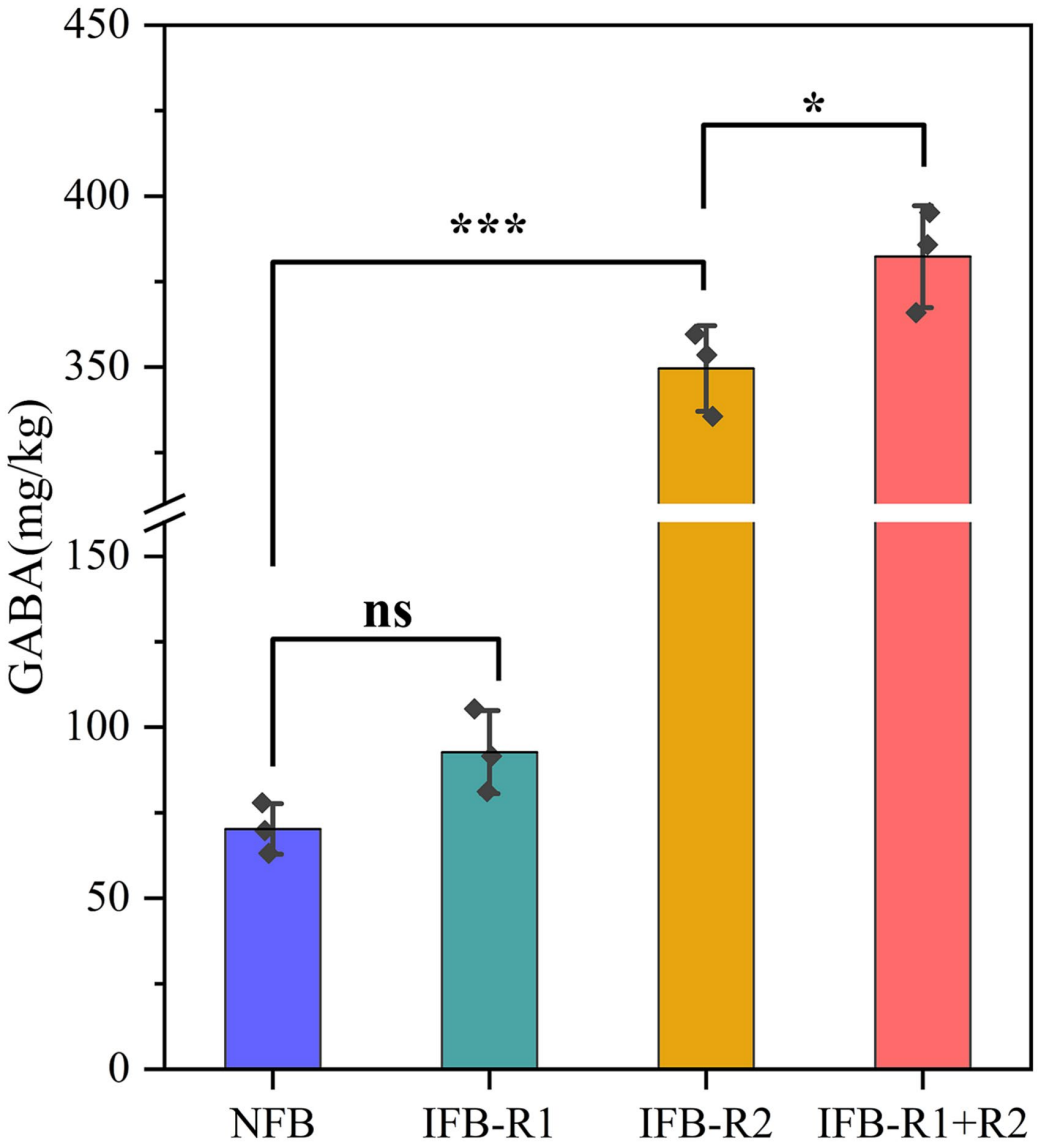
GABA content of bamboo shoots after 14-day fermentation. Significant differences are marked with asterisks between groups (“ns” means *p* > 0.05; “*” means *p* < 0.05; “***” means *p* < 0.001).

As shown in Figure 6, it was observed that the mono-inoculation of *L. plantarum* R1 did not lead to a significant increase in GABA content in bamboo shoots after 14 days of fermentation (*p* > 0.05). However, the introduction of *L. brevis* R2 alone resulted in an elevated GABA content of 349.60 ± 10.20 mg/kg, and the combined inoculation of *L. plantarum* R1 and *L. brevis* R2 further increased the GABA content to 382.31 ± 12.17 mg/kg. This represented an increase of over four times compared to natural fermentation (70.27 ± 6.04 mg/kg). Similarly, Moore et al. (2021)reported an enhanced concentration of GABA in cucumbers through the fermentation process, while Hirose et al. (2021) observed a substantial rise in GABA concentration in pickled luffa when inoculated with *L. brevis* (from 5.3 mg/100 g to 23.8 mg/100 g).

It is evident that the co-fermentation of the two strains greatly promotes GABA production. In a study focused on fermented milks, the co-culture of *Streptococcus thermophilus* and *L. brevis* demonstrated increased GABA production (Wu Q. et al., 2015). Similarly, another study showed that co-fermentation with *Leuconostoc mesenteroides* SM and *L. plantarum* K154 led to a notable increase in GABA content (Kwon et al., 2016). The production of GABA and the activity of GAD are intricately linked. Liu et al. (2023) concluded that the optimum pH for GAD is 3.6, while the ideal pH values may vary somewhat depending on the source of GAD, it generally functions more effectively under acidic conditions. We deduce that *L. plantarum* R1 creates a conducive acidic environment, thereby enhancing the activity and expression of GAD. At the same time, the substantial production of GABA contributes to the slight rise in pH during fermentation. The transport of glutamate for intracellular synthesis of GABA, facilitated by specialized transporter proteins, as well as the release of GABA into the extracellular environment, leads to the consumption of H^+^, resulting in an increase in ambient alkalinity (Cataldo et al., 2020). Furthermore, it has been proposed that traditional fermenters have a heightened efficiency in breaking down proteins into polypeptides. This provides *L. brevis* with ample nutrients to promote its growth. However, it should be noted that the precise mechanism by which GABA production is increased through the co-cultivation of *L. plantarum* R1 and *L. brevis* R2 still needs to be further investigated. In conclusion, the co-inoculation of *L. plantarum* R1 and *L. brevis* R2 for bamboo shoot fermentation emerges as an optimal approach to increase the GABA content in bamboo shoots.

#### 3.4.4 Nutritional composition of fermented bamboo shoots

The above experiments revealed that the co-inoculation of the two strains proved more advantageous for the fermentation of bamboo shoots compared to the mono-inoculation. To further investigate the nutritional qualities of the fermented bamboo shoots, protein, total sugars, reducing sugars, fat and crude fiber were analyzed. As shown in Table 4, the protein content of bamboo shoots increased during fermentation. NFB and IFB-R1 + R2 showed increases of 0.47 g/100 g and 0.66 g/100 g, respectively, compared to the fresh bamboo shoots (FBS). It was observed that inoculation with LAB strains in synergistic fermentation significantly enhanced the protein content (*p* < 0.05), consistent with findings by Singhal et al. (2021), Azeez et al. (2022), and Wang et al. (2023). Both total and reducing sugars decreased compared to the FBS, attributed to the microbial metabolic activities during fermentation, converting sugars into alcohols and organic acids. The fat content in bamboo shoots remained low both before and after fermentation, with a slight decrease after fermentation due to active microbial activities. Regarding crude fiber, the content increased from 13.92 g/100 g to 15.80 g/100 g and 16.58 g/100 g after natural fermentation and inoculated fermentation, respectively. Similar observations were made in other studies, such as oat bran, where crude fiber content increased from 14.36 to 15.83% through fermentation (Mustafa et al., 2022). Abdul Rahman et al. (2017) also found an increase in acid detergent fiber and neutral detergent fiber through fermentation. This suggests that fermented bamboo shoots are a promising option for low-fat and high-fiber foods.

**Table 4:** Changes in nutritional composition (g/100 g, fresh weight) of bamboo shoots before and after fermentation.

	Protein	Total sugar	Reducing sugar	Fat	Crude fiber
FBS	2.47 ± 0.04^c^	4 ± 0.02^a^	2.75 ± 0.04^a^	0.47 ± 0.04^a^	13.92 ± 0.03^c^
NFB	2.94 ± 0.07^b^	3.12 ± 0.01^b^	2.37 ± 0.04^b^	0.42 ± 0.02^ab^	15.8 ± 0.09^b^
IFB-R1 + R2	3.13 ± 0.03^a^	2.96 ± 0.02^c^	2.24 ± 0.02^c^	0.39 ± 0.02^b^	16.58 ± 0.04^a^

FBS, fresh bamboo shoots. Means in the same column with different superscript letters indicate significant differences among samples (*p* < 0.05).

#### 3.4.5 Sensory quality of fermented bamboo shoots

**Figure 7:**
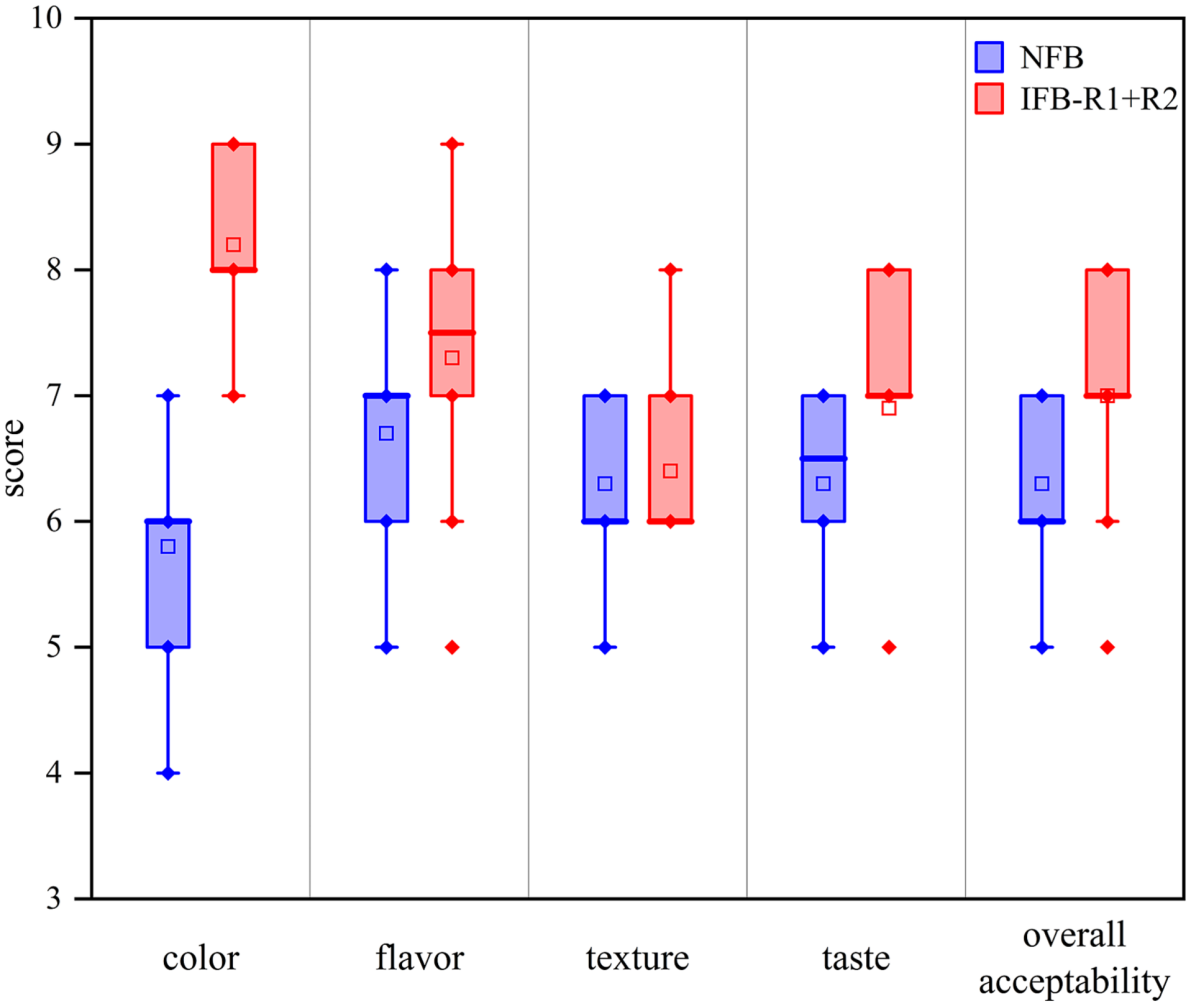
Sensory evaluation of bamboo shoots after 14-day fermentation.

To further investigate the sensory qualities of the co-fermented bamboo shoots, ten judges were invited to perform sensory evaluations. As depicted in Figure 7, a significant difference in color evaluation between NFB and IFB-R1 + R2 was observed (*p* < 0.05). However, there were no significant differences in flavor, taste, texture, and overall acceptability (*p* > 0.05). The uniform and creamy white coloration of IFB-R1 + R2 stood in stark contrast to the yellowish or partially browned appearance of NFB, which was the primary factor contributing to the higher scores. The rapid decrease in pH values during inoculated fermentation contributed to the degradation of lutein and chlorophyll, resulting in a lighter color. Conversely, the activities of enzymes like polyphenol oxidase and peroxidase appeared to increase the likelihood of browning in NFB. Regarding flavor and taste, the majority of judges noted that the acidity and freshness of IFB-R1 + R2 more closely resembled commercially available products. The short fermentation time for NFB had not yet allowed it to attain the desired level of acidity. Overall, after only 14-day fermentation, the inoculated fermented bamboo shoots exhibited superior sensory characteristics compared to naturally fermented ones.

## 4 Conclusion

In this study, *L. plantarum* R1 and *L. brevis* R2 were screened and identified as optimal strains for inoculating fermented bamboo shoots. The synergistic fermentation approach resulted in a substantial enhancement of GABA content in bamboo shoots compared to individual strain fermentations. Notably, this co-fermentation led to an impressive 4.5-fold surge in GABA content when compared to natural fermentation. Meanwhile, the nitrite content in bamboo shoots was reduced to a safe level of 5.96 mg/kg. Indeed, the fermentation process has led to an increase in protein and crude fiber contents and a reduction in fat content, which suggests that fermented bamboo shoots could offer a more nutritious and potentially healthier option. Sensory evaluations confirmed the superior flavor profile of co-inoculated fermented bamboo shoots. However, further optimization of the inoculation and fermentation conditions is crucial to achieve even higher quality bamboo shoots. Additionally, more experiments are warranted to validate whether GABA-enriched fermented bamboo shoots can offer enhanced health benefits. In summary, our research provides a novel strategy for bamboo shoot fermentation processing and offers fresh perspectives for the production of functional foods with health-promoting properties.

## Data availability statement

The original contributions presented in the study are included in the article/supplementary material, further inquiries can be directed to the corresponding authors.

## Author contributions

MC: Conceptualization, Data curation, Formal analysis, Investigation, Methodology, Software, Validation, Visualization, Writing – original draft. HX: Conceptualization, Funding acquisition, Project administration, Resources, Supervision, Writing – review & editing. XZ: Investigation, Methodology, Validation, Writing – review & editing. DT: Conceptualization, Resources, Validation, Writing – review & editing. HZ: Investigation, Methodology, Writing – review & editing. ZH: Investigation, Methodology, Writing – review & editing. AG: Conceptualization, Methodology, Project administration, Resources, Supervision, Writing – review & editing. JL: Conceptualization, Funding acquisition, Project administration, Resources, Supervision, Writing – review & editing.

## Footnotes

^1^ https://www.ncbi.nlm.nih.gov/
